# Points of Interest in the Care of Children’s Teeth

**Published:** 1896-11

**Authors:** B. J. De Vries

**Affiliations:** Holland, Mich.


### THE DENTAL REGISTER.

Vol. L.] NOVEMBER, 1896. [No. 11.

### PROCEEDINGS.

### Points of Interest in the Care of Children's Teeth.

BY B. J. DE VRIES, D.D.S., HOLLAND, MICH.

I will include under this subject children between the ages of four and fourteen, as that is the period of life that great depredations usually exist in the oral cavity.

The prevailing idea is that when a boy or girl has grown up to manhood or womanhood, then they must have their teeth looked after, as that is the period of life when selfrespect is generally asserted.

Young men and women call upon a dental practitioner sooner and oftener on account of their personal appearance than from a sense of the utility of the organs of mastication. Call to mind your appointment book and note your cases. Here is one who says: "I am going to have my teeth attended to. What can you do with this or that tooth? Is it worth saving? or shall I have a plate?" Or, perhaps, mother says: "I must have a gold filling in my front tooth." She wants nothing else.

There is another class of patients that are driven to our offices with the toothache, and we exert an influence on them that is beneficial for the preservation of these organs, for they heed our warning and have their teeth cared for and saved.

The deplorable condition the dentist is called upon to witness, the putrefaction, disease and suffering, both in young and old, can, to a large extent, be traced back to boyhood or girlhood, before the age of fourteen. Show me a boy or girl at the age of fourteen with all the teeth *in situ*, free from decay or properly filled, correctly articulated and properly cared for, and I will show you a man or woman who will point with pride to his dentist and who will be a blessing to his offspring and a comfort to himself.

Then, since our boys and girls of to-day twenty years from now will be our men and women, filling all vocations of life, it becomes evident that if we desire a race with well-cared for and sound teeth, *childhood* is the time to train, to regulate, to prune, before the destructive agencies or malpositions will stamp upon that face the marks of neglect of bygone days. The gardner deals tenderly with his young saplings as its top towers heavenward—he bends, prunes and cultivates that he may obtain a tree of symmetry and beauty whose shade will be sought by many a weary traveler.

We, as dentists, whose duty it is to care for children's teeth when diseased, to watch with great care the permanent teeth advance and take the places of the temporary teeth, have a higher duty to perform, a duty that may not bring us directly any dollars and cents, a duty that costs us nothing which we can perform as we are about our daily business—the education of our patients in regard to the dental organs.

Every mother ought to know that at the age of six, or thereabouts, the first permanent molar makes its appearance immediately back of the deciduous molar. She must be instructed about the value of this tooth during that critical period of childhood, that the second molar will not appear until the twelfth year, and that the child will have to depend upon this tooth, to a great extent, for mastication.

It is a universal rule that parents will mistake this tooth for a deciduous molar, yes, and even eight out of every ten physicians will make the same mistake.

Note the surprise which is manifested when we tear, with cold steel, an abscessed, ugly-looking molar from its hiding-place in the mouth of an eight-year-old child who could rest neither day nor night, whose moaning and earache, through reflex neurosis, perplexed both parents alike; and it remained an open question whether the child really had the mumps or whether a tooth might not have been the cause of all this suffering.

The first question after the removal of the cause is, " Doctor, will she get a new one for that?" "Where ignorance is bliss, it is folly to be wise," and if we think it will shock the parents too severely we can safely answer this question in the affirmative, for we know that the twelfth-year molar will get there some way, at all hazard, if it has to get there head foremost.

This total ignorance of the public in regard to the dental organs is deplorable and how best to educate our people in the care and preservation of the teeth is an open question to be solved by the dental profession of to-day. A comparatively small part of any community comes in contact with a dentist and have established habits of daily using the brush, and especially is this so among our children from four to fourteen years. Where parents do not enforce these habits, and interest themselves concerning the wellfare of their children how can we expect a better condition.

It is also true that in spite of all efforts some teeth will not stand the test of time, and we will not deny that a perfect and healthy set of teeth can exist in spite of all neglect. These are exceptions, however, and not the rule. Nature, unassisted, is as mysterious as nature, assisted, is wonderful. A child may pass through the period of eruption of the deciduous teeth without an ache or pain. These organs may perform their functions perfectly and in due time be replaced by permanent teeth as perfect and symmetrical as the former. But, alas! this is not always so. Too often the skill of the dentist is taxed to its ut most in arresting decay and preserving the beauty and symmetry of the face.

How, then, shall we educate the public along this line? The dental profession itself is inadequate to the task; so we must look elsewhere for aid. Where better can we go for assistance than to that grand educator itself—the public schools of our land. Already there has been added to the curriculum of our schools such branches as hygiene and physiology. Why not also give instruction, in a practical way, that can be comprehend by the young in the care of the teeth? If one hour in every week was devoted to this subject, and the subject rightly handled, it would be time well-spent.

Such questions as these taught from charts with few illustrations, would fill a long-felt want:

Give the number and names of the deciduous and permanent teeth. Time of eruption. What kind of food is beneficial to tooth structure?

How should teeth be brushed? With what? Etc., etc.

I believe, if some wide-awake dentist would write a small, attractive work on this subject to be used in the public schools, he would confer a great benefit upon society, and I am sure it would not be without remuneration to himself. Society must learn that a dentist can not save teeth without the assistance of their own efforts in caring for the same. There are those who will not, among men and women as well as children, but let us do something for those that know not how and why, who never come near our offices, and who are waiting to be taught that they may care for their own teeth as far as in their power lies, and to be able to discern when they need the assistance of a dentist.

We take it, then, for granted, that if we expect to see good results from our labors we must establish habits of cleanliness. The use of the brush and a proper antiseptic mouth-wash are essential parts to be performed by our young patients. And without these habits our work will be null and void.

On the other hand, the surgical and mechanical part to be performed by the dentist is of great importance.

Because the deciduous teeth are only for temporary service is no reason why they should not be properly cared for and filled. We can expect far better permanent teeth, and better development of the maxillæ where the deciduous teeth are well cared for and properly exercised, and it should be the aim of every dentist to give this subject the utmost attention however arduous the task may be.

The approximal surfaces of the deciduous molars are most frequently attacked by decay, and if not filled in time these spaces will form most acceptable places for microbes of fermentation and putrefaction. Of all suffering we are called upon to relieve, in nine cases out of ten, it is in this region, in connection with the first permanent molar.

It is my practice to fill these temporary teeth with cement only, and as soon as possible after decay has attacked them.

I never extract them if I can combat the difficulty in some other way. Very often I have found after much ulceration fungus growths upon the gums, and the apical portion of the roots exposed. In such cases, as also in the incisors, I nip off the exposed roots, leaving the crowns *in situ*, to fill up the space and favor the expansion of the maxilla.

I have heard of dentists devitalizing a deciduous tooth with arsenic and filling the roots with gutta-percha. I must confess that I never attempted such an operation on account of the difficulty in managing children, and the many obstacles we have to contend with in manipulating these delicate tissues.

Wherever I find it necessary to save a deciduous tooth, where the nerve is decomposed, I render it aseptic with peroxide and cloves and carbolic acid $\frac{1}{2}$ and $\frac{1}{2}$. After that I fill the roots with shreds of cotton impregnated with aristol dissolved in chloroform and fill the cavity with cement.

My reason for filling these roots with cotton is that it is next to impossible to apply enough hot air so as to get them perfectly dry, and if there should be any septic matter left in the canals, cotton will more readily absorb it, and the aristol will render it antiseptic.

What to do with the first molar is another perplexing question. A great many of our young patients have to lose this tooth at an early age. The best time to extract the same we will not here discuss but we will say, that many of them are extracted that should be saved.

This is the most useful and best adapted tooth for mastication of all the dental organs. Upon the loss or retention of this tooth hinges many intricate problems of irregularity and articu lation. When the structure of this tooth is fairly good, and if attacked by decay at all, the most favorable surface is invariably the anterior proximal; the reason for this can be readily seen when there is decay in the posterior proximal surface of the second deciduous molar. Food will be impacted between these two teeth, and through firmentation the first molar will be affected at the anterior proximal surface.

The question is often asked, should, in such cases, the tooth be filled with a permanent filling?

We believe that if the tooth structure is good, and there is a chance to make a perfect operation, we should not hesitate to fill this tooth with gold; but the chances are that we find decay at the seventh and eighth year, and the second deciduous molar will not be shed until the twelfth year which makes it difficult of access.

In such cases I fill this tooth with a phospate filling, and as soon as the second deciduous molar is shed, and before the second permanent bicuspid is erupted, I fill the first permanent molar with a permanent filling. This method obviates the necessity of cutting through the grinding surface, and in many cases can be accomplished without the rubber-dam. For, let us remember that all operations on the teeth of children should be conducted with the utmost of care and as painless as possible.

DISCUSSION.

The discussion of the paper presented by Dr. De Vries, in connection with the paper presented by Dr. Adams, was opened by Dr. Loeffler, as follows:

"The subject presented in Dr. Adams' paper is one that is being largely considered in the country just across the line—Canada; and it seems that they are getting the start of us, and it seems to me that, at this day, the closing days of the nineteenth century, we ought to do more than advance theories. Our profession is recognized by learned men outside of it as an exceedingly practical one; we are a practical profession, we have practical men—medical men admit that. Now, then, if our profession is known to be so practical, we ought to do something that is practical, and the ideas that are suggested here by Doctor Adams are those that I had in mind, and we ought to do something in this line here; we ought to do something to alleviate suffering, especially for children who have no means, perhaps, of alleviating their pain; we ought to do it in the way suggested by the reader of the last paper; we ought to instruct our people, and while we advance theories, we ought to take measures to do that. If we say that the public in general—the parents, ought to be instructed, we ought to see that the theory is carried out; we ought to see that the School Boards are instructed in that way—we should, at least, counsel with them. This matter should be brought before the medical profession, they have a great many patients—the same patients that we have—under their care; they, perhaps (because they are vastly in the majority), have occasion to see more than we. And, another way would be by means of lectures in our Public Schools; by means of appropriate articles in daily papers, or weekly papers if you please; magazines that are read largely. That would be a very favorable way of introducing this subject. But the idea that I wish to get at is, that it ought to be done. If it is for the betterment of our profession; for the uplifting of our profession, we ought to have hospitals in our larger cities, at least—we ought to have one in Grand Rapids, we ought to have one in Detroit where there are a great many children who would need that attention, and, as one of the speakers has suggested, on account of the ignorance that prevails our people, who ought to know better, do not, after they have been advised, give it the proper attention. It is simply because it has not been brought to their attention, perhaps, in the right way; it ought to be done in such a way that it will be carried out. There is nothing that would so instill this matter into the minds of the people of to-day as to have this practical operation carried out. Let us have the children's teeth examined in school; we have them examined for every other purpose. They learn every other conceivable thing—why not learn something that is practical, something that is to their own welfare and for their own good? This is something they ought to learn—they may not think so, but it is simply because it has not been compulsory, or because it has been put to one side.

Dr. Hoff: I fully sympathize with both the papers that have just been read, and with the remarks of Dr. Loeffler. He says we must do something. We can not, in the United States, compel people to do things by law or by rule. We can persuade them to do some things, and in this way our effort will have to be made. For this reason, any efforts in the line of legislation may be slow work, but we can do something to create a public sentiment in favor of hygienic instruction in our schools and adequate supervision of the health of the inmates of our schools and institutions of various kinds. There is no question that if the dental profession was in earnest and active in this matter, that much might be done to bring about a public sentiment that would demand from our law-makers some enactments that would bring about a work of this character. If we can not accomplish this through our laws, perhaps we can induce benevolent people to establish hospitals, or we ourselves can become more benevolent than we have been. I see no reason why every dentist in Grand Rapids should not be willing to serve for one or two days in the year, or twice a year, on a committee to examine the teeth of the children in the Public Schools. I think that an examination, conducted in this way, would be much more effective than one that is made covering the entire year, or the entire six months by a single person. Perhaps it would not be so thoroughly or systematically done, but it would put the whole Public School system in your city in mind of the fact that they have teeth, and that those teeth are diseased, and that they need treatment, and many of these children would go home to their parents and tell them that the dentist had been to school to-day, and he tells us our teeth need filling, and those children would hunt up their dentist, if they were able to afford one, and if they were not they would seek out these benevolent institutions where such work might be done gratuitously. In this way it seems to me something might be done for the benefit of the public without any law and without any very great effort on the part of the practitioner. This might be done in every town where there is one dentist, or where there are two or more dentists. Every town and village in our State might be, in this way, organized into an Examining Board—voluntary, but none the less effective. If this Association were to pass a resolution calling upon the dentists of the State to take up this matter and make it a business for a year or two at least, until we could demonstrate whether there was anything to be gained by such work.

I do not think there can be any danger of transmitting diseases of the mouth in our public institutions from the children coming in actual contact with each other; such diseases are not transmitted because of contact—they are not contagious, and the air that the children exhale, while it may contain noxious particles of various kinds, is comparatively inoffensive if the children be in reasonably good health, because this air that is exhaled (because of its specific gravity) is not again inhaled by the children immediately. It seems to me that many infectious diseases are not the result of actual contact of the children as they associate on the play-ground and with one another in their classes, but from the filthy condition of the room and the building in which the children are housed. In this climate almost every one is afflicted with catarrhal affections of varying degrees, there is more or less sputum deposited on the floor of these buildings, carrying with it more or less offensive material from diseased teeth, mouth and throat, which dries on the floor, rises in dust and contaminates the air of the whole room. The poor children are affected, but no more, perhaps, than those of the more prosperous class—the children of our best families go to the Public Schools. They are all in the same room, and all are subjected to the same conditions. Oftentimes the children of families in better circumstances are the least able to resist these disease-producing conditions, and they fall a prey to the offensive conditions that surround them more quickly than some of the poorer children who live outdoors more, or have better air in their homes, for their homes are not so close and tight, or are not kept so warm and unsanitary. As a matter of self-preservation we ought to take up this subject; we can not afford to neglect it. It seems to me that if the doctor would make his plea from this standpoint that he would make more progress, and would get more sympathy; that he would enlist a more hearty co-operation. If we want to undertake this matter we must take it up from this standpoint rather than from the legislative, for we can hope for very little from direct legislation at the present time.

We must educate the public to the necessity for it; we must educate our physicians. Our medical brethren do not appreciate this matter nearly so much as we do; they do not think of it, their attention has never been called to it. When medical men look in the mouth all they see is the tongue, they never see the teeth, they never see any of the conditions of the mouth except the tongue. If the tongue is coated, or has a certain condition, it indicates a certain systemic condition; they do not look any further; they say, "It is not my business to look after the teeth, the dentist will do that." Very many times there are conditions of the mouth that are not manifest on the tongue at all to them, but that we, as dentists, see and recognize more readily than a medical man can. The only fault with us is that we have been too modest; we see so much of these conditions that we become rather hardened. We do not all feel that it is an important condition, and perhaps we do not see it in its worst form. If we were to establish a hospital-infirmary, where we might get at some of the poorer class of people who do not take any care of their teeth at all, perhaps we would see more of these bad conditions. I think it is high-time that something were done in this way, that some notice should be taken of the offensive and contaminating conditions of the mouths of the children in our Public Schools.

That other matter which Dr. De Vries touched upon—the loss of the first molar teeth—is one of the greatest importance. He says that he *sometimes* extracts that tooth; he seems to think that it is not a very objectionable thing to do. I have almost come to the conclusion that never should a first molar tooth be extracted, particularly in the lower jaw; it should be retained in the mouth as long as it is possible in almost every case. The headlong position of which the second molar teeth take in trying to fill up the gap left from the extraction of the first molar leaves a deformity in the articulation which is very serious. It deprives our patients not only of the use and benefit of the one tooth that is extracted, but practically the whole set of molar teeth are lost for mastication. Perfect mastication can not be performed in a mouth where the first molar teeth have been lost, and the various forms of injury will result from such extraction. Too much importance can not be attached to early attention to this tooth and its salvation by whatever means—whether it is filling or whatever it may be—whatever can be done to prevent its decay should be done, and that right early.

DR. FOX, of Ironwood: Mr. President, these two papers have brought out thoughts that are almost inexpressible. I have been situated in a locality that has brought to mind these same thoughts that have been suggested here to-day, a great many times. I live in the iron-country, where all of the extracting of teeth has been done by the physicians, as a rule. The laboring classes there pay a dollar a month for their physician, which is deducted from their wages, and they have one physician—the mining physician. The people there are of all nationalities that are on the face of the globe, and it is very humiliating to a dentist to examine the mouths of some of those people who have lived in the mining district for many years, and to see the deformities and the wholesale slaughter of teeth that has been carried on in the copper and iron-districts of Michigan.

DR. MCCLOUD, of Ironwood, was on the Board of Education and I saw the necessity of some movement of this kind, and I called attention to the fact some two years and a half ago. The School Board had a meeting, and at that meeting they asked me if I would take it upon myself to deliver two lectures during each term of school to the children on the care of teeth. I accepted, and told the doctor that I would gladly do it for the benefit of those suffering little children, as has been mentioned in these valuable papers 18th P.M.S. that have been read here. Dr. McCloud also made arrangements with me to do the extracting for the hospital, and he would give his patient a note to me, and I have tried to do a missionary work in the way of educating the people to take care of their teeth; and when these papers were read I was glad to know that others were thinking about this matter, but I hope there are not many of you placed in the same position that I am, because if you are you will turn away in shame from extracting many times, and I only hope that in every county, in every town in the State of Michigan, and all the States, they will adopt some measure for the care of the teeth in the Public Schools as well as all institutions controlled by public charity and otherwise.

Dr. Harvey, of Battle Creek: I think that the members of this body are all representative men of the communities in which they reside, they all have more or less influence with the municipal officers of the governments of the towns and cities in which they live, and the results to be obtained through any efforts which we might propose to make, would depend upon individual effort to a very large extent; yet I believe that there should be some decided action by this body in the way of resolutions, that the individual dentists might have something to refer to that would give weight to his arguments. We can go before a School Board and show the benefits that could be derived from the course to be pursued, as has been outlined here, yet it would give more weight to it if we had some action taken by this Association.

I, for the last two years, have had upon my roll a membership of two hundred worthy-poor children whose teeth I have cared for; every one of those children come to my office twice a year; about one hundred and twenty-five of them are from a charitable institution. I do the work free, and the condition of their health generally has improved very much, although their medical care is great. I instruct these children how to clean their teeth, and with that, I take a great deal of pains, and I see good results from my efforts.

I trust that there will be some decided action taken before we adjourn finally.

Dr. Root, of Hart: A short time ago I changed from a city to a country-practice, and while in the city I refused to extract teeth that I could save, but I have changed my mind; I am one here to-day to say that I extract teeth, and the main reason why I do it is, that if they can not afford to pay me I can not afford to work upon them. We may talk upon theory, but the practical part of it is: What can we do for the patients, and what can the patients do for us?

Now, take a six-year-old molar that comes to you, and they can not afford to have you treat it. Can you go ahead and do it out of your own pocket? I don't know how it is with the rest of the people, it takes about all I can do to get around without throwing in some extra time. I used to think that I was willing to give quite a good deal of time to saving people's teeth, and if anybody came into the office and didn't have the money it was all right. I would go ahead and perform the operation because I thought they ought to save their teeth; that was the feeling I had when I left college. We were taught that we should take particular pains in saving all the teeth that came to us, and it was a kind of humanity-scheme that we were going into, but I realized after I got outside for a while that we are all working for a living.

In this city I had the great privilege of working under this first idea that came into my head while in college, and I filled the teeth of quite a few people, spent a good deal of time on them; they were poor, and I took my chances, because their teeth looked bad and I thought I ought to do it. Another thing: I said, Why I can save this tooth for you, and I won't pull it! And they would go away. Now, what is the practical use of all this? We have got to educate these people before we can do this; we have got to educate every one of them—not only to come to us twice a year and have their teeth examined, but to pay properly. You have all noticed in your practice that the majority of the children are, in the beginning, afraid of you. I have had quite a few cases come to me where the little child was perhaps not two years old, and was greatly frightened when it came into the office, just from what it had heard the parents say, and wept all the time it was there. Now, if these children were educated in the first place, even if they were poor, if they were not told at home how it was going to hurt them, they would naturally want to come to you and have their teeth taken care of, whether they were poor or not. They will work around and earn the money to do it. In the place where I am I have quite a few of the boys around town whose parents are poor, and all of them are doing job work and every little thing they can do to have their teeth taken care of, because they have been educated to it and know that it is right.

I am not in favor of a hospital for the benefit of the poor class in the dental line. I don't think it is right; it seems to me no one will appreciate a thing you do for them, unless they do something themselves in return for it. You hardly ever find people so poor in this country but what they can give you a little something for taking care of their teeth.

I was very much interested in the paper presented by Doctor Adams, and agreed in many points with it. And while we are theorizing here, how shall we do this; shouldn't we get some practical plan as to how this work should be accomplished? Take a child six or seven years of age, with temporary teeth, where there are little abscesses breaking out in the gums and they come to you. What would be the advice of the average dentist here, as to what to do with those—extract them? They are past filling; there is no use filling them. What would you do, extract them and let the place go together, or leave them and let them keep on abscessing?

Dr. White, of Benton Harbor: Dr. Adams' paper I have listened to with very much interest, and also that of my brother from Holland, and Dr. Hoff's remarks. I believe that we are touching the key-note when we are talking about starting in at two years old and educating the children to take care of their teeth. I do not think that we would lose that six-year-old molar very many times if we could get hold of the child at two years of age, as suggested by Dr. Adams, and take care of that child's teeth twice a year until he is fourteen years of age.

I believe that this talk and these papers are in the right direction, and that if we could spare at least one hour in the week to devote to the care of the teeth of poor children it would be a benefit to them and an honor to our profession.

Dr. Harvey: I wish to reply to Dr. Root. I think he reckons without his host when he says that we do not get pay for doing work for the worthy-poor. I do not think that there is any dentist who can do work for the worthy-poor and not be rewarded for it, and he doesn't wait until he gets up above either. I know that I have not, and I know that I can spend a great deal of my time in that way, and I can be rewarded for it in a pecuniary way, though I do not look for it.

Dr. Hoff: Dr. Root's remark brings an idea to mind that might be fruitful also. He says that he has not time to give to this kind of work; I presume there are a great many dentists who feel as though every hour and every minute of their time was worth money to them, and so it is. Many of us work after hours; we work more than ten hours a day, but there is help to be had; there is help that is unemployed; there is help that would be glad to serve in some capacity in this connection. We have, for instance, in the University every year one hundred and fifty or more undergraduate students who have a three months' vacation to spend, and there is not a week but what I have from one to two or three, or perhaps a half a dozen inquiries as to whether I know of any dentist who needs help; they want to get into an office where they can get office-experience; they want to learn something that no dental college—I do not care how thoroughly equipped or organized it is—can teach. We can not teach practice of dentistry as dentistry is practiced in an office, and students know this, and all of you know it. Students need this perhaps more than anything else, they need some kind of office-experience, and it seems to me that here is an opportunity where these undergraduates, and, perhaps, some of the recent graduates, can be utilized to advantage. Some of you gentlemen who have more to do than you can attend to can afford to pay these men something; you can, at least, afford to pay their board, or if you can not do that, you can take them into your home and take care of them in the summer during the three, or five, or six months that they are out of college. Many of them would like some place where they could go and simply get their board or their living and have an opportunity of doing some kind of dental work and getting an office-experience, something that would be valuable to them and which they could use when they get out into practice. You could use them upon the work of these poor children, and they can do their work for you; they can polish your rubber-plates just as well as you can; they can grind teeth just as well as you can; they can vulcanize teeth; they can keep your books; they can collect your bills, and occasionally they can clean teeth for these little ones just as well, and if they don't know how, in an hour's time, or two hour's time well spent to instruct them how to clean these teeth thoroughly and you can utilize them, and you can do these little ones a favor and at the same time do these young men a greater favor, perhaps, than the children, and you will relieve yourselves of much of the drudgery of your office business. These young men would all be glad to do it, they are strong and vigorous and they are anxious to work, and they want places to work, but as it is now they go out into the harvest-field or they go to selling books; some of them loaf all summer and have nothing to do, but every one of them would be glad to go into an office where he can be useful; somewhere where he could learn something; somewhere where he could get an idea of how business it conducted in the dental practice; how a dentist handles patients—that is something we can not teach in a dental college. We handle patients in the infirmary in a wholesale way, and not in a retail way. You can take these young men into your offices and do them a service, and it seems to me you can utilize them in this way and do them a favor, and there are many things in this subject of the treatment of children's teeth wherein you can utilize them just as well as you can use yourselves, and I hope we will have more inquiries at the University and at the Detroit College for students to work in your offices and to assist about the office in the future.

Dr. Porter, Petoskey: Mr. President and Gentlemen—I can not see how it is possible for those of us who reside in places, for instance, like Petoskey, of five or six thousand inhabitants, to do anything else than partially, at least, charitable work. We can not help it. We have done a great deal of it in Petoskey, and as has been mentioned, a great deal of that kind of work may be done by an assistant. I happen to be fortunate enough to have a very good one, and a young lady assistant also, who is very efficient, after having been properly trained. It seems to me that in towns of that size we can not help doing a great deal of charitable work, if we do our work conscientiously, as we must do it in our profession if we live up to what we ought to be. There are, as has been mentioned, a great many poor people whose children suffer; for instance, I know of more than one widow in our town who supports a family of, anywhere, from three to eight children, and those children, the majority of them, are small, from two years up to eight or ten, and it is impossible, as you well know, for such people to pay for anything that is not positively necessary, in fact, their medical bills have to go unpaid unless they are paid in some charitable way. Of course, there are provisions for medical assistance in almost every town and State in the Union, but not for any dental assistance. There is a defficiency that we can see which the people at large do not see. The course that I have endeavored to take in our own town has been to educate the people, as has been mentioned, through the public schools by a lecture occasionally, and the publishers of daily and weekly papers are very willing to publish short articles occasionally, and it is not necessary to monopolize the thing, or have our names published at all, but only to put the matter before the people so as to gradually educate them to what we think they ought to be in order to appreciate the situation.

Dr. A. W. Diack, Detroit: It seems to me that this subject is a great deal deeper than anything we have touched upon during this discussion; in fact, so deep that we can not enter into it any more than we have done or do it as well as we should like. In the practical application of the theories that have been brought out, in so far as they are applicable to the State of Michigan, it seems to me that spreading this information among the poor children is going to be very slow indeed, and it is not going to be accomplished through any legislative acts which the dental society might cause to be enacted; it is to come rather through the personal influence of the dentists, through the chartable organizations, and then through an organization which, I think, needs education in dentistry almost as much as any other organization, namely, the medical profession. I think there is a great deal of influence in the profession, and if we could once direct it to pay proper attention to the condition of the teeth, it would be our most powerful ally in this educational effort.

And in order to have some standing or some authority behind us as persons or as practitioners of dentistry, in this matter,

I think it would be proper for this society to pass resolutions to the effect that there should be some legislative enactment or, at least, that some action should be taken looking to the estab- ment of such an act, and in that way follow out the idea brought forward by Dr. Harvey. I think that is the best manner in which we can arrive at a definite conclusion of the matter.

Dr. Hoff: Before we adjourn I move that the chair ap- point a committee of five to draft resolutions in accordance with the sentiment of this meeting as expressed this afternoon and present them tomorrow afternoon at 4:30 P. M., at our business meeting, for the consideration of this body.

Dr. Diack: If Dr. Hoff will accept an amendment to that motion, I move that a permanent committee on resolutions be appointed, as I think, in looking over the programme, there will be other questions requiring resolutions to be passed, and if that commitee would be made permanent during this session, I think, it would be a little more in order, and would facilitate business.

The motion of Dr. Diack was carried.

The meeting here adjourned.

EVENING SESSION—JUNE 10, 1896.

The meeting was called to order by President House.

The minutes of the previous session were read by the Secre- tary, and approved.

The Chair appointed, as a Committee on Resolutions, Doctors Field, Parker, Loeffler, Harvey and Diack.

A paper on "The Absorption of the Root of the Temporary Teeth," by Dr. A. W. Haidle, of Detroit, was here read by Dr. Diack. (See August Register, page 388.)

Dr. N. S. Hoff opened the discussion of the above paper as follows:

Mr. President and Gentlemen: I feel myself wholly un- able to discuss this paper intelligently and to any purpose, and you will see now the reason why I was anxious to have Dr. Taft here when this paper was read; Dr. Taft has done soms work along this line, and has some ideas about it that would be worth a great deal more than anything I can say.

In the first place, I have to say that I can not agree with the position that the paper has taken. I don't know that I ought to take this position; I certainly can not take it from any definite knowledge, because I have done little or no experimental work in this direction, but, on general principles, I do not see how the process of absorption of the roots of the teeth can be accomplished in the manner indicated in the paper. The old theory or the commonly accepted theory that acids play an important part in the removal of the roots of the temporary teeth it seems to me is a rational one. The great difficulty, of course, is for us to make any experiments that will identify the acid and show its connection with the process. It is exceedingly difficult to make experiments in the living tissues, particularly of a human being. We can not experiment upon each other at liberty, and particularly in this connection, and there is no opportunity in the animal world for making these experiments with any degree of accuracy, and it is almost impossible for us to determine what the nature of the solvent is in this connection.

The chief reason why I conclude that it must be an acid is that we have no other solvents that will produce such rapid solution of hard, bony structures, particularly such structures as the enamel of the teeth, or even the bone which is more soluble. We have no solvent which will produce a similar amount of solution in the same time. This solution of the roots of the temporary teeth is often very rapid in its progress, and I don't know of any solvent that will produce this solution in the same way and to the same extent and with the same rapidity. I am free to say that I do not know how this acid is generated. It may be the result of the continued irritation of the growth of the new tooth in the tissues, causing destruction of the soft tissues which underlie the tooth, and in this way a viscid acid secretion may be generated in the cells which surround the tooth, which sets up a fermentative or putrefactive process, in which the acid is formed. In what is termed "an acid condition," where the acid is newly-generated, it is more active in dissolving the bone, and in this form no one would question its power to act upon the lime-salts of the tooth; of course it would not dissolve the matrix, the organic matrix; that would have to be absorbed by the absorbent vessels around about the tooth, and we can readily explain this from similar digestive process in other parts of the body.

Now, the other theories that have been advocated and advanced it seems to me need very little consideration, because we all know that it is practically impossible to dissolve bone, ivory, or bony structure, such as we have in the root of a tooth, in any of the ordinary fluids of the body. These fluids are alkaline; sometimes they have a slight acid reaction in diseased conditions, but ordinarily they are alkaline, but not sufficiently alkaline at any time to produce any very great absorption of bone, and particularly of the root of a tooth, so I can not see that there is any fluid in the tissues—in the circulation or in the tissues themselves—that it would ever be possible to dissolve a tooth in.

The effect of pressure from a growing tooth—I can not see either how that would have anything whatever to do with the absorption. It might cause considerable congestion, and perhaps inflammation around about the tooth, and we might have a hypernutritive condition as a result of the increased blood-supply: that, possibly, might have an excessive absorbent quality, but not sufficient, it seems to me, to account for the amount of solution that occurs.

Now as to this theory that Dr. Haidle advances, that the solution of the root of the tooth is due to the fact that some kind of a digestive fluid is formed or is generated in this tissue and attacks the tooth and digests it, I can not very well harmonize this with the ordinary digestive processes. If we undertake to account for the process on this theory, we must have organs that are capable of secreting a digestive fluid, a fluid that will digest the bone or the root of a tooth. We have organized tissue about the teeth, but this little carneous body that we all recognize and see in the root of these dissolving teeth is not an organized structure at all, it is simply a change of the connective-tissue cells, a collection of them in which they are oftentimes of a pathological condition or form rather than of a physiological form, but they certainly are not organized in such a way that they would secrete a ferment such as is secreted by the glands of the [illegible]mentary canal.

In the next place, we know there is no organized body of this kind in this tissue, because of the fact that when we make sections of this tissue we do not find any nerve supply, there is no nervous mechanism; there are sensitive nerves, of course, distributed in that system, and there may be motor-nerves distributed to the blood-vessels, but there are none that can be traced to these different cells as Dr. Black has differentiated them. He has simply given them different names, according to the functions which they evidently perform, but there is no difference in the histiological construction of them so far as I have been able to determine from his description of them. I think they are simply a modification of the same kind of cells, or the same kind of tissue, and because they happen to be in a location where a certain process takes place, he gives them a certain name. They certainly have no such organization as we find in the peptic glands which secrete that digestive fluid, or in the other glands of the stomach or the other digestive organs, and there is no nervous mechanism there that can be stimulated to excite these glands to secrete a digestive fluid; and if there was any ordinary digesting fluid that we are familiar with in the digesting process would not be able to digest the tooth or roots of a tooth, or the hard, bony structure of the root of a tooth as rapidly as we know it takes place in the absorption of these temporary or deciduous teeth.

Dr. Haidle seems to be of the opinion that there is a ferment secreted here, not an organized ferment of a putrefactive kind, but a digestive ferment—something that sets up a fermentation in these tissues. I can not see how this analogy can be reconciled here. He takes his idea, I think, largely from the fact that Dr. Miller found in his investigations, in his work on the manner in which the teeth decay, that an acid was the result of a putrefactive influence that takes place there. In this condition we do not have putrefaction, we do not have suppuration, we may have a certain kind of suppuration, but it never goes on to putrefaction, and we never have any cause for the formation of acid which will cause a solution of the tooth such as is produced in the decay of a tooth—we do not have the same phenomena. We do not find the tooth absorbed in the same way that decay of the tooth is produced. We do not have what is sometimes called or recognized as the white decay or the rapid decay or the brown decay, or the black decay—those various kinds of decay that we used to think came as the result of the kind of acid that was generated and caused the solution of the tooth ; we do not have any of this phenomena. Of course this condition is quite similar to that decay of the tooth or that abrasion of the tooth which occurs under the influence of lactic acid, or those abrasions of the teeth that we sometimes find cut across the faces of the teeth, and we have attributed this kind of abrasion or decay to the lactic acid that is generated in the mouth, but we are not certain ; and Dr. Miller himself is not certain that that particular variety of decay is the result of fermentation.

So I can not see, in looking at this subject from my standpoint, that there can be any special, organized body or glands there that would secrete any formation that would have the power or the ability to absorb the roots of the teeth in that way. It seems more reasonable to me and more rational that it is the result of the formation of an acid in the formentative process.

Fortunately, I had a little patient come in a few days ago with two or three temporary teeth which I removed, and this carneous body came away with one of them, in a mass. I gave it to our histologist to make some slides for me that I might see just what that body is, to see if there was anything like an organic tissue, but he was very busy, and he didn't get the slides made for me, so I didn't have an opportunity to examine them, but he assured me that he did not think that there could be any special organ in that tissue. He said he thought it was very improbable, and so I think myself that there is no special organ there that will eliminate a peculiar ferment, for it must be peculiar ; it must be different from any of the digestive ferments that we know anything about that would cause this solution of the tooth. So, for lack of evidence, I, for one, am compelled to believe and hold to the old idea that it must be an acid of some kind. Just how that acid is generated, or what kind of an acid it is, I don't know, and this idea, while it is new, is incapable of demonstration. I do not see how it can be demonstrated, or how we can get any data on such a hypothesis. It seems to me it must be clearly hypothetical, and that, therefore, we can not prove it.

Dr. Corbin, St. Johns: While I have never made any experiments to determine the question at issue, I have had occasion to observe many times conditions that might throw some light on the question—they directed by thoughts and conclusions in a certain channel. We all know that where the vitality of the pulp is destroyed in the permanent teeth, so-called, where the crowns have been rotted away for a long term of years, that the roots grow shorter, that the roots will be absorbed from the apex end, and they certainly will, in the course of years, be thrown entirely out of the jaw by that process, if left alone in healthful jaws. I think it is the physiological condition that does the work, and that, of course, is independent of pressure. We have all seen the deciduous teeth in the mouths of people thirty, forty or fifty years old—probably there is not a dentist here of ten year's practice who has not seen it—it is a very common thing to see the baby teeth in the mouths of adults in the place of the teeth that should have replaced them, and by the side of the teeth that should have replaced them. I have seen a great many cases, in my almost forty year's experience, of the temporary canines, and sometimes bicuspids, standing exactly in line with the other teeth of the arch, more especially in the lower jaw, and sometimes in the upper jaw, and the tooth that should have replaced them entirely missing—never had made their appearance at all.

It is only a few years since this association convened at Ann Arbor, and there are doubtless many persons here now who were present then and remember something about a case of a good deal of interest, and that had given much trouble to the physicians of a family in Pontiac—a woman thirty or forty years old. It was then transferred to the care and charge and investigation of some dentists in Detroit, and the woman was finally brought to Ann Arbor and presented at the session held there of this association, and various dentists present examined the left side of the lower jaw back; the third molar, I think, had never made its appearance, and there had been trouble, and it was finally diagnosed that it had never been erupted, by a number of dentists, and some one, there, in the presence of the association, succeeded in extracting that tooth. Now, there was a case that was forty years old and had grown to full size and had never been erupted nor made its appearance at the surface of the gum.

I think, in cases where I have seen the temporay teeth standing exactly in line with the permanent teeth of the second set, and in place of the tooth that had never made its appearance, that that tooth doubtless had grown in the wrong direction, and the deciduous tooth had remained, while I used to think the reason it remained was because it had not been pressed upon by the growth of the second set of teeth, and, hence, absorption had not occurred. Now, in the case to which I alluded a moment ago, where these deciduous teeth stand, in addition to all the second set of teeth, exactly in line, or nearly in line, the permanent tooth has been deflected from its natural course and grown up closely in contact by the side of this deciduous tooth. In the mouths of people thirty or forty years old, and younger, twenty or twenty-five years old, I have had occasion to extract such teeth under similar circumstances and by pressure draw back the permanent tooth into its proper place, and wherever I have extracted these teeth I have found the absorption, if they were closely in contact, of the whole length of the roots of the deciduous tooth, one-half of it, one-third of it, or two-thirds of it, just simply to give place to that portion of the permanent tooth that came up closely in contact with it or had crowded it; the absorption had taken place, not from the end entirely, but from the side of the whole length of the root, apparently in response to the pressure of the growth of the permanent tooth.

We do know that the roots of the permanent teeth are absorbed and shortened and the acid theory might account for the destruction of the tooth if that destruction commenced at the surface of the gum, if it commenced where acid could be applied, but I can not see how acid can be applied to the end of the roots of the permanent teeth; I can not see how it can be applied to the ends of the roots of baby teeth in healthful condition, the surrounding soft parts being protected.

Dr. Hoff: It is generated there—it is not applied.

Dr. Corbin: I am not prepared to dispute that, but then it is a little difficult for me to see how the acid can be generated and applied down in the gum, in the midst of a healthful circulation. It may be, but I don't know how, and almost invariably you will find, I think—all of you who will refresh your recollection on the subject—that where you have seen the deciduous teeth remaining until late in life it is where the permanent teeth have never put in an appearance, where either the germ did not exist or did not grow, or grew in the wrong direction and where there was no pressure in consequence; at least that has been in accordance with my observations.

Dr. Loeffler, of Saginaw: I have given this subject some study, and I am inclined to take the position that Dr. Corbin has. It seems to me that we might theorize on this subject as well as with any other subject that can only be handled in that way. We know that in various parts of the body, by pressure certain things are absorbed; we know that the root of the permanent tooth, or of the temporary tooth for that matter, is absorbed in case of abscesses. Now what is it that causes the absorption in that case? Most of us understand that it is the pressure caused by the formation of pus in that part. Now, if it is pressure in that case, is it not barely possible that the pressure caused by the growth of the permanent tooth causes the absorption of the root, due to the fact that we have enamel in contact with the dentine or the cementum of the root, that is, wouldn't it be fair to assume this? As the last speaker has intimated we find, and I have found it that way in many cases, that when, for any reason, the permant tooth does not come, or is not directly under the root of the temporary tooth, we find that this absorption takes place along the side, all the way along, and perhaps one half or one-third or even two-thirds of that tooth is absorbed. Now, is it not barely possible on account of the growth in the one case, at the expense of the structure before it—and it may, in this case, be a physiological process nevertheless, that on account of this pressure, as I say, the tooth before it is absorbed. And, as has also been mentioned, where we do not find the temporary tooth, or where the permanent tooth is missing, the root of the temporary tooth is in every case left as it is, we do find any absorption. Now why it should be that, in this case, the carneous body or whatever term it may have is wanting in that case, can not be accounted for. Therefore, it seems to me that this theory would, at any rate, be one that ought to have a place.

DR. DIACK, of Detroit: When Dr. Haidle asked me to read his paper I was very much inclined to the acid theory regarding this phenomena, but since reading it and looking over the subject I have come to the conclusion that so far as I can see, with the observations that I have made, that he has, really, to my mind, assumed the only rational solution to this problem.

Regarding the acid theory: To my mind I can not reconcile it with the fact that the serum of the blood in which this dissolution of the temporary roots take place is alkaline; any acid which comes in contact with the alkalinity of the blood must be overcome by it; you can not have an acid acting on any substance, such as the roots of the temporary teeth is, in an alkaline form.

That, I think, disposes of the acid theory.

Regarding the supposition that an acid is formed, to my mind, it seems hardly necessary to go outside the natural law that we see in existence in the human body. The only acid we have secreted for any physiological function, not for any function of excretion, such as uric acid and the other acids that are thrown off, but the acid that is secreted for a physiological function is the hydrochloric acid of the stomach, and that acts only in a secondary manner, it assists the pepsin—the pepsin can act only in an acid solution. Therefore, bringing up the idea of another secretion of an acid for the mere absorption of these temporary roots, it seems to me, is going out of the way. I see no reason for it.

Dr. Hoff has spoken of organs set aside for the absorption. I think that this giant cell that Dr. Black has mentioned, the myloid cell and the large cell with the many nuclei, can be taken as the digestive organ; we see its presence in every absorption of tissue that takes place in the body.

In so far as the nervous influence is concerned, the mere fact that we have not yet discovered, microscopically, the presence of any nerve running to this part does not, in my mind, establish the fact that the theory of the nerve influence is wrong, because in the fœtus we can not discern, with the microscope, any nerve influence acting upon the fundamental cells of the fœtus; I think that the same thing takes place there; it is merely physiological, and is governed by a power that we have no idea of whatever.

Then, again, in the absorption or the digestion, as we have been pleased to term it, in the roots of the temporary teeth, it seems to me there can be a digestive fluid formed by this organ, as I have termed it, that would be capable of dissolving the lime salts in the root. The lime salts, of course, consisting of the calcium carbonate and the other salts, if it can not be brought to the tooth in solution there must surely be some cell which will cause it to be in solution in the first place before it can be absorbed into the body, and if that is the case, the opposite can also be true. So that we can have these multi-nucleated cells absorbing this calcium carbonate and other mineral salts from the dentine and from the cementum. That, to my mind, is satisfactory.

Then, again, another argument for the idea or theory that it is a digestive process that takes place rather than a solvent one. A glance at the shape of the root of a temporary tooth I think will satisfy any one—at least it satisfies me—that it is due to digestion rather than solution by an acid, because the part of the dentine that immediately surrounds the living pulp in a temporary tooth always seems to be more persistent, it has more resistant power to the process, whatever it is, that is going on; and you all know that while any living organism may be dissolved by the gastric juices, that it takes a longer time to digest a living tisssue than it does one that is not living, whereas if an acid were acting it would be acting as an acid, and would have no effect whatever and there would be no difference in effect whatever, whether it were on a dead or living tissue; it would be calcium carbonate just the same, and the acid would be working on it just the same—you would have the ordinary chemical reaction.

In regard to the presence of this carneous body, I think that it is nothing more or less than the result of irritation and stimulation, a hypertrophy of the tissues, or the giant cells in contact with the temporary teeth. To my mind, this idea brought out by Dr. Haidle is more satisfactory than the acid theory.

Dr. Porter: There is another thing that is a little unexplainable, and that is the want of effect upon the roots of the deciduous teeth, providing the crowns of such teeth decay by either the acid or digestive theory. That is something that has always been a puzzle to me and I suppose is to all of us, why the roots of the deciduous teeth are not affected by either of these theories if the crown of the tooth be decayed, and also (which complicates it even more) if we fill the roots of the deciduous teeth, and in that way keep the crown perfect, that is, keep them from further decay, the roots of the deciduous teeth will, after that, absorb as naturally apparently as they would had the pulp remained alive.

